# Food Insecurity in a Sample of Informal Caregivers in 4 Southern US States

**DOI:** 10.5888/pcd19.220069

**Published:** 2022-08-18

**Authors:** Swarnali Goswami, Siddhi Korgaonkar, Kaustuv Bhattacharya, Meagen Rosenthal

**Affiliations:** 1 Department of Pharmacy Administration, School of Pharmacy, University of Mississippi, University, Mississippi

## Abstract

**Introduction:**

Given the disproportionate burden of food insecurity in the southern US states and the high prevalence of caregiving in this area, we assessed caregiving-related predictors of food insecurity among caregivers in 4 southern US states.

**Methods:**

We used data from the 2015 Behavioral Risk Factor Surveillance System (BRFSS) for individuals aged 18 years or older who resided in Alabama, Louisiana, Mississippi, and Tennessee to assess the association between caregiving status and food insecurity, accounting for the complex survey design of BRFSS. Caregiving-related predictors of food insecurity were identified by using multivariable logistic regression.

**Results:**

Weighted counts of caregivers and noncaregivers were 356,198 and 652,737, respectively. Prevalence of food insecurity was higher among caregivers than noncaregivers (35.9% vs 25.9%). Adjusting for sociodemographic predictors, caregivers had 56% (95% CI, 1.30–1.87; *P* < .001) higher odds of food insecurity than noncaregivers. Among caregivers, those caring for a spouse or a partner (adjusted odds ratio [aOR] = 1.7; 95% CI, 1.02–2.85; *P* = .04) had significantly higher odds of food insecurity compared with those caring for parents or parents-in-law. Caregivers who had been caregiving for 6 months to 2 years had higher odds of food insecurity compared with those who had been caregiving for less than 6 months (aOR = 1.88; 95% CI, 1.12–3.16; *P* = .02). Caregivers who reported a need for support services had higher odds of food insecurity compared with those who did not (aOR = 3.38; 95% CI, 2.19–5.21; *P* < .001). Caregivers caring for people with musculoskeletal conditions, compared with people with neurologic conditions, had higher odds of food insecurity (aOR = 3.47; 95% CI, 1.52–7.91; *P* = .003).

**Conclusion:**

Caregiver screening for food insecurity in health care settings and linkage to appropriate food and caregiving support resources should be prioritized by future health policies.

**Summary**

**What is already known on this topic?**

Southern US states bear a disproportionate burden of food insecurity and also have a higher prevalence of informal caregivers compared with other US states.

**What is added by this report?**

The characteristics of caregivers associated with food insecurity have not been examined previously, so we assessed caregiving-related predictors of food insecurity among caregivers in Alabama, Mississippi, Tennessee, and Louisiana.

**What are the implications for public health practice?**

Screening of caregivers for food insecurity in health care settings and subsequent linkage to appropriate food and caregiving support resources should be a priority of future policies targeting food insecurity.

## Introduction

Informal caregivers provide unpaid assistance or supervision with personal tasks not including childcare to a relative or friend who cannot perform these tasks because of cognitive, physical, or psychological impairments (1). Southern US states were reported to have the highest prevalence of informal caregivers (≥25%) during 2015 to 2017 (2). Caregiving is demanding and is associated with poor health outcomes such as chronic stress, obesity, diabetes, and mental health problems (3–5). Additionally, age-adjusted rates of informal caregivers reporting fair or poor health in Alabama, Louisiana, Mississippi, and Tennessee during 2015 to 2017 were reported to be 20% or more (2). Caregiving has been recognized as a public health issue, and its burden is likely to worsen with the rapidly growing aging population in the US (6).

In addition to adversely affecting caregiver health, caregiving also creates financial strain on the caregiver, which could affect their ability to afford food. Approximately 20% of a caregiver’s income is reportedly spent on caregiving expenses, with household and medical expenses being the biggest drivers of caregiving-related expenses (7). A 2012 study reported that caregivers were twice as likely to report food insecurity compared with noncaregivers (8). Between 2017 and 2019, the household food insecurity rate in southern US states was higher than that of the rest of the country (9).

Despite southern US states bearing a disproportionate burden of food insecurity and caregiving, the characteristics of caregivers associated with food insecurity has not been examined. We assessed the prevalence of food insecurity among adult caregivers and the association of food insecurity and caregiving status in 4 southern US states: Alabama, Louisiana, Mississippi, and Tennessee. We also sought to identify caregiving characteristics associated with food insecurity among caregivers. Our findings will help plan appropriate policies for assisting caregivers most at risk of food insecurity.

## Methods

This was a cross-sectional study of data from the 2015 Behavioral Risk Factor Surveillance System (BRFSS) that included adult informal caregivers in Alabama, Louisiana, Mississippi, and Tennessee.

The BRFSS is a collaborative project of the Centers for Disease Control and Prevention (CDC) and the US states and territories. BRFSS data are collected annually from noninstitutionalized US adults by state-based surveillance systems following a population density–based strata sampling design and random-digit–dialing telephone survey. Each respondent is assigned weights calculated through iterative proportional fitting for each stratum to be considered nationally representative. Because BRFSS data are publicly available, this project was deemed exempt from institutional review board review. We used data from 2015, the latest year for which the information on both caregiving and food insecurity was available in the BRFSS data set.

### Measures

Adult informal caregivers were identified from the survey item, “People may provide regular care or assistance to a friend or family member who has a health problem or disability. During the past 30 days, did you provide regular care or assistance to a friend or family member who has a health problem or disability?” Those who replied yes or no were included in the analysis, and their caregiver status was designated as such. Those who refused to answer the caregiving question were excluded from analysis.

Food insecurity, which is the lack of reliable access to affordable and nutritious food, was assessed via the item, “How often in the past 12 months would you say you were worried or stressed about having enough money to buy nutritious meals?” Those who responded “rarely” or “never” were considered food secure, and those who responded “always,” “usually,” or “sometimes” were considered food insecure. The food insecurity variable was dichotomized following the methodology of previous studies (10). Respondents who had missing values for the food insecurity question were excluded from the analysis.

Caregiving characteristics considered for examining predictors of food insecurity among adult informal caregivers were relationship with caregiver (parent or parent-in-law, child or grandchild, spouse or partner, other), care recipient condition (mental or neurologic, metabolic or cardiovascular, musculoskeletal, cancer, or other), caregiving 40 hours or more per week (yes or no), months spent caregiving (less than 6 months, 6 months to up to 2 years, 2 years or more), need for support services (yes or no), helping with activities of daily living (ADLs) and instrumental activities of daily living (IADLs) (yes or no). ADLs are personal activities that a care recipient might need an informal caregiver’s help with and were identified from the BRFSS item, “In the past 30 days, did you provide care for this person by managing personal care such as giving medications, feeding, dressing, or bathing?” IADLs are activities that are broader in scope, requiring coordination and planning, with which the care recipient might need an informal caregiver’s help (11), and were identified from the BRFSS item, “In the past 30 days, did you provide care for this person by managing household tasks such as cleaning, managing money, or preparing meals?” Informal caregivers often benefit from support services such as classes about caregiving activities (eg, giving medications, help in getting access to services, support groups, individual counseling to help cope with giving care, respite care) that enable them to take better care of their care recipients. Therefore, need for support services was assessed from the BRFSS item, “Of the following support services, which one do you MOST need, that you are not currently getting?” Responses were dichotomized into needed any type of support services or did not need any.

Sociodemographic variables included age, sex, race and ethnicity, education, marital status, employment status, annual household income, health insurance, and Metropolitan Statistical Area (MSA) indicator. We controlled for MSA in our analysis because it had been reported that the prevalence of food insecurity in urban areas is higher than in suburban or rural areas (9).

### Statistical analysis

**Figure 1:**
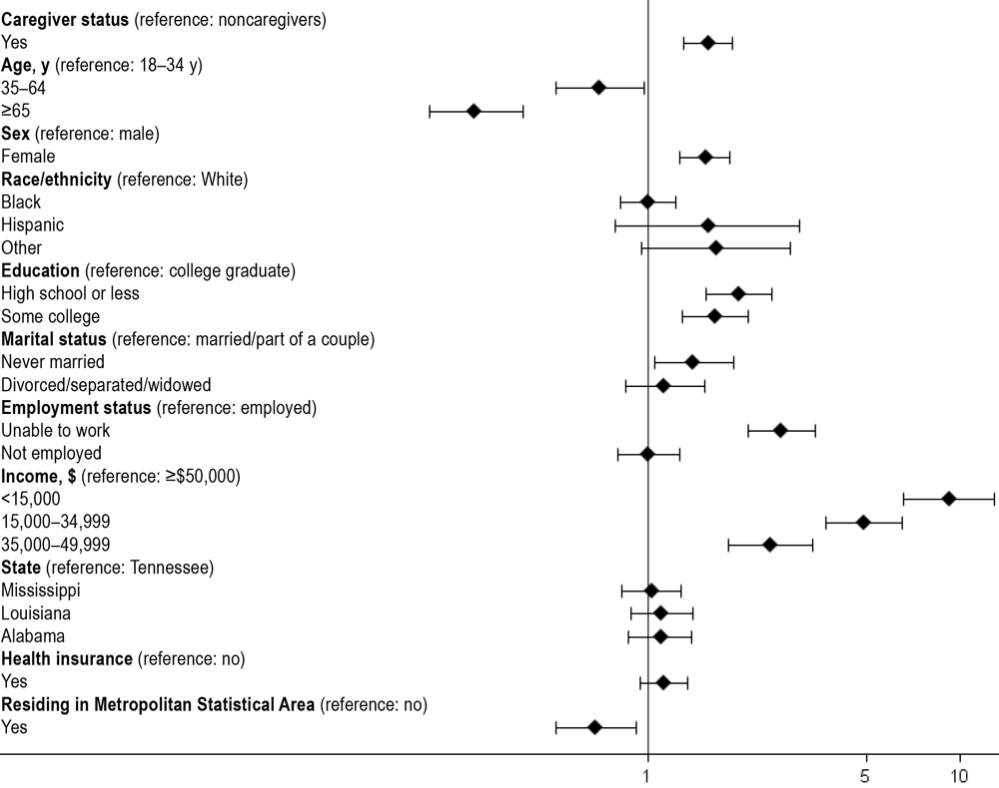
Association of caregiver status with food insecurity, adjusting for sociodemographic covariates, Behavioral Risk Factor Surveillance System, 2015.

**Figure 2:**
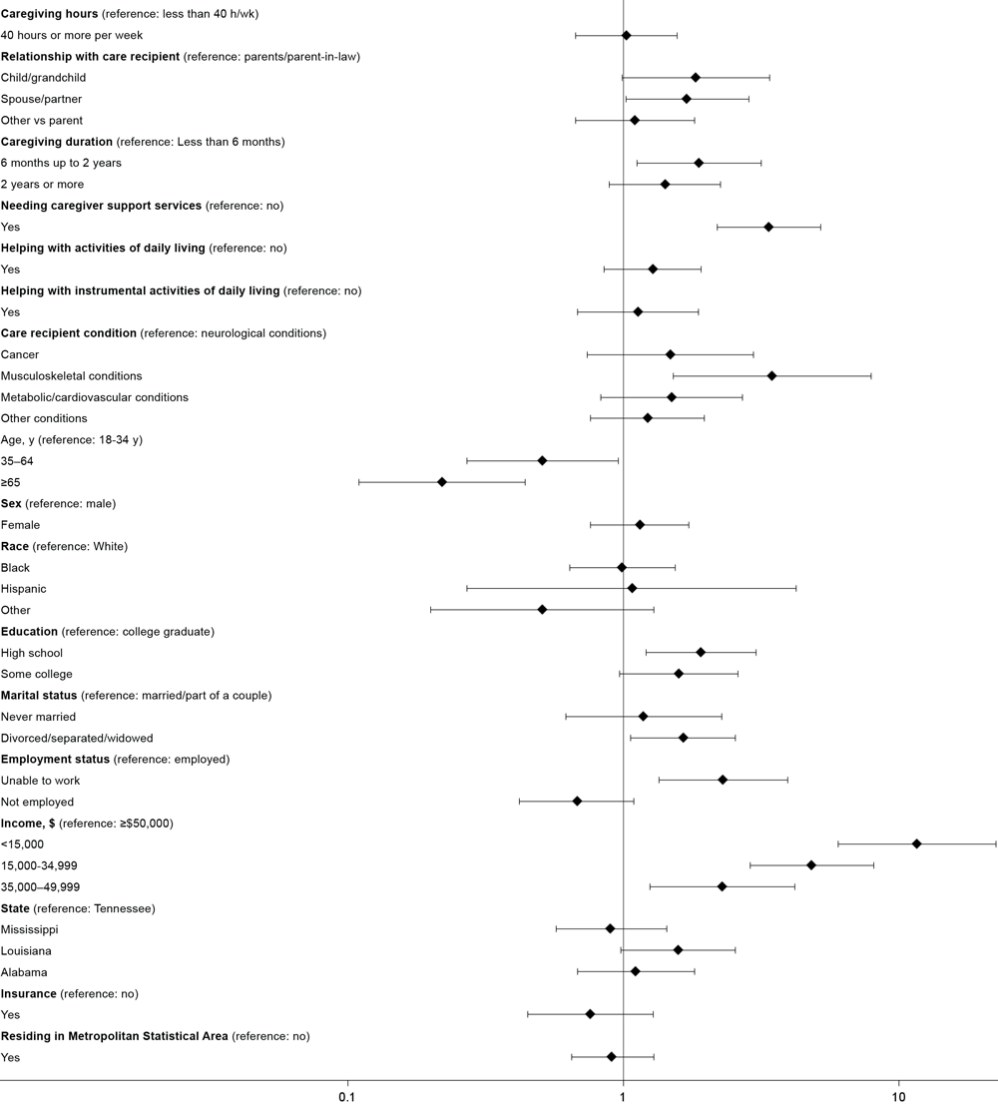
Association of caregiver characteristics with food insecurity, adjusting for sociodemographic covariates, Behavioral Risk Factor Surveillance System, 2015.

We described the overall and state-based prevalence estimates of food insecurity among caregivers and noncaregivers using weighted percentages, and we described the sociodemographic characteristics as proportions, by caregiving status. We also reported the caregiving-related characteristics of the caregivers in the sample and the prevalence of food insecurity among them. The association between caregiving status and food insecurity was determined by using a multivariable logistic regression model, adjusted for sociodemographic characteristics (Figure 1). Sociodemographic and caregiving-related correlates (relationship with caregiver, care recipient condition, hours per week, months since caregiving began, need for support services, assisting with ADLs, and assisting with IADLs) of food insecurity were assessed by using multivariable logistic regression (Figure 2). We reported odds ratios, 95% CIs, and the associated *P* values. All analyses accounted for the complex sampling design of the BRFSS, and appropriate subsample procedures and survey weights were used. Analysis was conducted by using SAS version 9.4 (SAS Institute, Inc), and complete case analysis was done for all analyses. We set significance at *P* < .05. 

**Table:** 

Characteristic	Adjusted odds ratio (95% confidence interval)	*P* value
**Caregiver status (reference: noncaregivers)**		
Yes	1.56 (1.30–1.87)	<.01
**Age, y (reference: 18–34 y)**		
35–64	0.70 (0.50–0.97)	.03
≥65	0.28 (0.20–0.40)	<.01
**Sex (reference: male)**		
Female	1.52 (1.27–1.83)	<.01
**Race (reference: White)**		
Black	1.00 (0.82–1.23)	.95
Hispanic	1.56 (0.79–3.06)	.20
Other	1.65 (0.96–2.85)	.07
**Education (reference: college graduate)**		
High school or less	1.96 (1.53–2.49)	<.01
Some college	1.64 (1.29–2.09)	<.01
**Marital status (reference: married/part of a couple)**		
Never married	1.41 (1.05–1.88)	.02
Divorced/separated/widowed	1.13 (0.85–1.52)	.40
**Employment status (reference: employed)**		
Unable to work	2.67 (2.09–3.42)	<.01
Not employed	1.01 (0.80–1.27)	.95
**Income, $ (reference: ≥$50,000)**		
<15,000	9.20 (6.59–12.85)	<.01
15,000–34,999	4.90 (3.71–6.49)	<.01
35,000–49,999	2.47 (1.81–3.38)	<.01
**State (reference: Tennessee)**		
Mississippi	1.03 (0.83–1.28)	.78
Louisiana	1.11 (0.88–1.4)	.37
Alabama	1.10 (0.87–1.38)	.42
**Health insurance (reference: no)**		
Yes	1.13 (0.95–1.34)	.18
**Residing in Metropolitan Statistical Area (reference: no)**		
Yes	0.68 (0.51–0.92)	.01

**Table:** 

Characteristic	Adjusted odds ratio (95% confidence interval)	*P* value
**Caregiving hours (reference: less than 40 h/wk)**		
40 hours or more per week	1.03 (0.67–1.57)	.90
**Relationship with care recipient (reference: parents/parent-in-law)**		
Child/grandchild	1.83 (0.99–3.39)	.05
Spouse/partner	1.7 (1.02–2.85)	.04
Other	1.1 (0.67–1.81)	.71
**Caregiving duration (reference: less than 6 months)**		
6 months up to 2 years	1.88 (1.12–3.16)	.02
2 years or more	1.42 (0.89–2.25)	.14
**Needing caregiver support services (reference: no)**		
Yes	3.38 (2.19–5.21)	<.001
**Helping with activities of daily living (reference: no)**		
Yes	1.26 (0.85–1.91)	.24
**Helping with instrumental activities of daily living (reference: no)**		
Yes	1.13 (0.68–1.87)	.65
**Care recipient condition (reference: neurologic conditions)**		
Cancer	1.48 (0.74–2.97)	.27
Musculoskeletal conditions	3.47 (1.52–7.91)	.003
Metabolic/cardiovascular conditions	1.5 (0.83–2.71)	.17
Other conditions	1.23 (0.76–1.97)	.40
**Age, y (reference: 18-34 y)**		
35–64	0.51 (0.27–0.96)	.04
≥65	0.22 (0.11–0.44)	<.001
**Sex (reference: male)**		
Female	1.15 (0.76–1.73)	.51
**Race/ethnicity (reference: White)**		
Black	0.99 (0.64–1.54)	.98
Hispanic	1.08 (0.27–4.23)	.92
Other	0.51 (0.2–1.29)	.15
**Education (reference: college graduate)**		
High school	1.91 (1.21–3.02)	.01
Some college	1.59 (0.97–2.60)	.07
**Marital status (reference: married/part of a couple)**		
Never married	1.18 (0.62–2.27)	.61
Divorced/separated/widowed	1.65 (1.06–2.55)	.03
**Employment status (reference: employed)**		
Unable to work	2.3 (1.35–3.94)	.002
Not employed	0.68 (0.42–1.09)	.11
**Income, $ (reference: ≥$50,000)**		
<15,000	11.61 (6.00–22.48)	<.001
15,000–34,999	4.83 (2.88–8.08)	<.001
35,000–49,999	2.29 (1.25–4.19)	.007
**State (reference: Tennessee)**		
Mississippi	0.9 (0.57–1.44)	.67
Louisiana	1.58 (0.98–2.55)	.06
Alabama	1.11 (0.68–1.81)	.67
**Insurance (reference: no)**		
Yes	0.76 (0.45–1.28)	.30
**Residing in Metropolitan Statistical Area (reference: no)**		
Yes	0.91 (0.65–1.29)	.60

## Results

Overall, 35.9% (95% CI, 33.9%–37.9%) of caregivers and 25.9% (95% CI, 24.8%–26.9%) of noncaregivers in Alabama, Louisiana, Mississippi, and Tennessee reported food insecurity in 2015 (Table 1). In these 4 states, the prevalence of food insecurity among caregivers was highest in Louisiana (38.2%; 95% CI, 34.2%–42.3%). Caregivers aged 18 to 34 years and 35 to 64 years had a higher proportion of food insecurity than their noncaregiving counterparts (18.1% vs 13.2% and 66.6% vs 60.3%, respectively), and they also had a higher proportion of food insecurity than caregivers aged 65 years or older (15.3%) (Table 2). Most caregivers who experienced food insecurity were aged 35 to 64 years (66.6%; 95% CI, 61.0%–72.1%). Among both caregivers and noncaregivers, most food-insecure individuals were White and female. Most of the food-insecure adults in the caregiver sample were either married or part of a couple (46.1%; 95% CI, 40.5%–51.6%) followed by those who were divorced, separated, or widowed (31.8%; 95% CI, 26.8%–36.8%). Unemployed adults reported a higher prevalence of food insecurity (38.0%; 95% CI, 32.4%–43.6%) than employed adults (36.1%; 95% CI, 30.7–41.5). Among the food-insecure caregivers in the sample, the highest proportion had an annual household income between $15,000 and $35,000 (46.7%; 95% CI, 41.2%–52.3%), had health insurance (74.9%; 95% CI, 69.5%–80.4%), and lived in an MSA (67.9%; 95% CI, 63.1%–72.7%).

**Table 1:** Prevalence of Food Insecurity in 4 Southern US States, by Caregiving Status, Behavioral Risk Factor Surveillance System, 2015

State	Prevalence of food insecurity, weighted % (95% CI)		
	Caregivers (weighted n = 356,198)	Noncaregivers (weighted n = 652,737)	*P* value^a^
Overall	35.9 (33.9–37.9)	25.9 (24.8–26.9)	<.001
Alabama	35.3 (32.1–38.5)	25.9 (24.1–27.6)	<.001
Louisiana	38.2 (34.2–42.3)	25.9 (23.7–28.2)	<.001
Mississippi	34.9 (30.8–38.9)	29.3 (27.2–31.4)	.01
Tennessee	35.3 (31.0–39.6)	24.0 (21.8–26.2)	<.001

a Calculated by using χ^2^ test.

**Table 2:** Sociodemographic Characteristics of Food Insecure Adults in Alabama, Louisiana, Mississippi, and Tennessee, by Caregiving Status, Behavioral Risk Factor Surveillance System, 2015

Characteristic	Proportion of food insecure adults, weighted % (95% CI)		
	Caregivers (weighted n = 356,198)	Noncaregivers (weighted n = 652,737)	*P* value^a^
**Age, y**			
18–34	18.1 (12.7–23.6)	13.2 (10.2–16.3)	<.001
35–64	66.6 (61.0–72.1)	60.3 (56.6–63.9)	
≥65	15.3 (12.2–18.4)	26.5 (23.6–29.4)	
**Sex**			
Female	66.4 (60.8–71.9)	66.6 (62.9–70.4)	.94
Male	33.6 (28.0–39.2)	33.3 (29.6–37.1)	
**Race and ethnicity**			
White	64.8 (59.2–70.3)	63.8 (60.2–67.4)	.20
Black	31.1 (25.6–36.5)	29.9 (26.5–33.2)	
Hispanic	1.9 (0.4–3.4)	1.4 (0.5–2.4)	
Other^b^	2.3 (0.9–3.7)	4.9 (2.9–6.8)	
**Education**			
High school or less	63.3 (58.2–68.4)	65.3 (61.8–68.8)	.38
Some college	28.8 (24.1–33.6)	25.5 (22.2–28.8)	
College graduate	7.9 (5.8–9.9)	9.2 (7.5–10.9)	
**Marital status**			
Married/couple	46.1 (40.5–51.6)	46.3 (42.4–50.2)	.07
Divorced/separated/widowed	31.8 (26.8–36.8)	37.4 (33.9–40.8)	
Never married	22.2 (16.6–27.7)	16.3 (13.4–19.3)	
**Employment**			
Employed	36.1 (30.7–41.5)	28.4 (24.8–31.9)	.04
Unemployed	38.0 (32.4–43.6)	39.7 (35.9–43.6)	
Unable to work	25.9 (21.3–30.6)	31.9 (28.5–35.3)	
**Annual household income, $**			
<15,000	28.4 (23.2–33.6)	28.2 (25.1–31.4)	.83
15,000–34,999	46.7 (41.2–52.3)	45.7 (41.8–49.6)	
35,000–49,999	10.4 (6.6–14.2)	12.5 (9.8–15.2)	
≥50,000	14.5 (10.2–18.8)	13.6 (10.6–16.6)	
**Health insurance**			
No	25.0 (19.6–30.5)	13.8 (10.8–16.8)	<.001
Yes	74.9 (69.5–80.4)	86.2 (83.2–89.2)	
**Metropolitan statistical area**			
No	32.1 (27.3–36.9)	31.8 (28.6–34.9)	.91
Yes	67.9 (63.1–72.7)	68.3 (65.1–71.4)	

a Calculated by using χ^2^ test.

b “Other” includes American Indian, Alaska Native, Asian, Native Hawaiian, Pacific Islander, or multiracial individuals.

Most caregivers were caring for their parents or parents-in-law (42.9%; 95% CI, 39.5%–46.3%), were caring for less than 40 hours each week (73.6%; 95% CI, 70.6%–76.7%), and had been providing care for 2 years or more (56.4%; 95% CI, 53.1%–59.7%) (Table 3). In terms of caregiving conditions, 18.9% (95% CI, 16.4%–21.4%) were caring for people with mental or neurologic conditions, 13.9% (95% CI, 11.8%–16.0%) for metabolic or cardiovascular conditions, 6.8% (95% CI, 4.8%–8.8%) for musculoskeletal conditions, and 7.8% (95% CI, 5.8%–9.7%) for cancer. Approximately two-thirds of caregivers (62.0%; 95% CI, 58.8%–65.2%) helped their care recipient with ADLs and 84.5% (95% CI, 82.3%–86.8%) with IADLs. Approximately one-fifth of caregivers (18.7%; 95% CI, 16.1%–21.3%) expressed a need for caregiver support services (Table 3). Prevalence of food insecurity was highest among caregivers who were caring for children or grandchildren who had a health condition (37.2%; 95% CI, 27.7%–46.7%) and those with musculoskeletal conditions (48.3%; 95% CI, 32.6%–63.9%). Food insecurity was also highest among those who were caregiving for 40 hours per week or more (39.2%; 95% CI, 32.3%–46.1%), had been caregiving for 6 months up to 2 years (34.0%; 95% CI, 25.5%–42.6%), expressed a need for caregiver support services (49.5%; 95% CI, 41.8%–57.3%), and were helping with ADLs (34.6%; 95% CI, 30.2%–39.1%) or IADLs (32.7%; 95% CI, 28.9%–36.3%).

**Table 3:** Caregiving-Related Characteristics and Food Insecurity Prevalence Among Caregivers in Alabama, Louisiana, Mississippi, and Tennessee (Weighted N = 356,198), Behavioral Risk Factor Surveillance System, 2015

Caregiver characteristic	Proportion of caregivers, weighted % (95% CI)	Prevalence of food insecurity, weighted % (95% CI)
**Relationship with care recipient**		
Parent/parent-in-law	42.9 (39.5–46.3)	28.9 (23.6–34.3)
Child or grandchild	10.7 (8.7–12.6)	37.2 (27.7–46.7)
Spouse/partner	19.5 (17.1–21.9)	29.6 (23.5–35.8)
Other^a^	26.9 (23.8–30.0)	33.6 (26.9–40.3)
**Care recipient health problems**		
Mental or neurologic	18.9 (16.4–21.4)	29.6 (22.8–36.4)
Metabolic or cardiovascular	13.9 (11.8–16.0)	33.4 (25.6–41.3)
Musculoskeletal	6.8 (4.8–8.8)	48.3 (32.6–63.9)
Cancer	7.8 (5.8–9.7)	33.2 (18.8–47.6)
Other^b^	52.6 (49.2–55.9)	28.7 (24.5–32.9)
**Caregiving 40 hours or more per week**		
No	73.6 (70.6–76.7)	28.3 (24.6–32.0)
Yes	26.4 (23.3–29.4)	39.2 (32.3–46.1)
**Months spent caregiving**		
Less than 6 mos	25.4 (22.6–28.2)	24.0 (18.8–29.3)
6 mos to 2 y	18.2 (15.5–20.9)	34.0 (25.5–42.6)
2 y or more	56.4 (53.1–59.7)	33.5 (29.0–37.9)
**Need for caregiver support services**		
No	81.3 (78.7–83.9)	26.9 (23.3–30.6)
Yes	18.7 (16.1–21.3)	49.5 (41.8–57.3)
**Helping with ADL**		
No	38.0 (34.8–41.2)	25.6 (20.9–30.3)
Yes	62.0 (58.8–65.2)	34.6 (30.2–39.1)
**Helping with IADL**		
No	15.5 (13.2–17.7)	23.3 (17.2–29.4)
Yes	84.5 (82.3–86.8)	32.7 (28.9–36.3)

Abbreviations: ADL, activities of daily living; IADL, instrumental activities of daily living.

a Other relationships include grandparents, siblings-in-law, other relatives, and nonrelatives/friends.

b Other diseases include asthma, chronic obstructive pulmonary disease, substance abuse, addiction, HIV infection, or organ failure.

After accounting for age, sex, race and ethnicity, state, income, education level, marital status, insurance status, and MSA, caregivers had higher odds of reporting food insecurity (adjusted odds ratio [aOR] = 1.56; 95% CI, 1.30–1.87*; P < .*01) than noncaregivers (Figure 1). Caregivers who cared for a spouse or partner (aOR = 1.7; 95% CI, 1.02–2.85*; P = .*04) had significantly higher odds of food insecurity compared with those who cared for parents or parents-in-law (Figure 2). In terms of caregiving duration, compared with caregivers who had been caregiving for less than 6 months, those caregiving for 6 months to up to 2 years had significantly higher odds of food insecurity (aOR = 1.88; 95% CI, 1.12–3.16*; P* = .02) (Figure 2). Compared with those who did not express a need for caregiver support services, caregivers who expressed a need for such support services had higher odds of food insecurity (aOR = 3.38; 95% CI, 2.19–5.21; *P < .*001). Finally, in terms of caregiving conditions, compared with caregiving for neurologic conditions, caregivers caring for people with musculoskeletal conditions, especially arthritis (aOR = 3.47; 95% CI, 1.52–7.91*; P *= .003), had higher odds of food insecurity.

## Discussion

In 2015, food insecurity was higher among caregivers, both overall and in the 4 southern US states we assessed — Alabama, Mississippi, Louisiana, and Tennessee — with the highest prevalence reported in Louisiana. Furthermore, caregivers in these states had higher odds of food insecurity even after accounting for sociodemographic characteristics. We found that caregivers who care for their spouses or partners and those who care for their children or grandchildren had higher odds of food insecurity than those who cared for their parents or parents-in-law. This finding could be due to care recipients such as spouses, partners, and children sharing the same household, which could result in increased health care spending, increased financial strain, and resultant decrease in resources to afford nutritious food (7). Food insecurity issues among caregivers of children with certain health conditions has been well documented (12,13). Although literature on food insecurity among those who provide care for spouses and partners is scarce, evidence exists of a substantial caregiving burden among caregivers for spouses with chronic or terminal diseases and its association with health conditions such as depression and anxiety (14,15). Therefore, caring for spouses or partners could result in worse physical and mental health of the caregivers, which in turn could increase health care spending and predispose them to food insecurity. Thus, screening for food insecurity should be made available for those caring for young children and for spouses or partners. Future food insecurity interventions should also prioritize such caregivers and their households.

According to our study, people caregiving for 6 months to less than 2 years were more likely to experience food insecurity than those caregiving for less than 6 months. This finding indicates that food-related stress may be more intense from 6 months to less than 2 years of the caregiving, a time that the caregivers would most require food-related support. Thus, new caregivers should be screened in the health care setting using validated food insecurity questionnaires and connected to appropriate food access programs such as the Supplemental Nutrition Assistance Program (SNAP), the Special Supplemental Nutrition Program for Women, Infants, and Children (WIC), and the Child and Adult Care Food Program (CACFP), and others (16). Validated food insecurity questionnaires have been the most widely implemented and evaluated method of screening in health care settings and have been reported to be effective (16). These resources will improve newer caregivers’ awareness of food access programs and make them better equipped to manage their food-related needs as they progress toward the more intense caregiving periods (17). Moreover, our study indicated that those who expressed a need for caregiver support services had a higher likelihood of reporting food insecurity. Informal caregivers tend to support their care recipients in managing symptoms, administering medications, changing bandages, and other medical and nursing tasks for which they often do not receive necessary training. Literature suggests that informal caregivers do not receive adequate support in medical care training and access to health care facilities, counseling, and support groups to cope with caregiving stress as well as respite care services (18,19), emphasizing the need for interventions that include provisions for connecting caregivers to appropriate channels where they can access such services (20). Addressing the unmet needs of these caregivers can help alleviate their financial strain, reduce caregiving-related mental and physical burden, and ultimately improve their food security. As of 2022, several federal and state-based health insurance programs such as Medicare Advantage and Medicaid cover a variety of in-home care services and nonskilled needs, such as help with daily activities, to promote aging in place (21,22). Alabama, Louisiana, Mississippi, and Tennessee have Medicaid programs that aid informal caregivers. In Alabama, Medicaid programs such as the Elderly and Disabled Waiver, the State of Alabama Independent Living (SAIL) Waiver, and the Personal Choices Program and Alabama Community Transition (ACT) Medicaid Waiver aid with home care. Louisiana has several Medicaid programs such as Long-term Personal Care Services Waiver, the Adult Day Health Care Waiver, and the Community Choices Waiver, which provide similar assistance. Mississippi Medicaid’s Elderly and Disabled Waiver provides a variety of in-home support and care services to individuals, including personal care and adult day care. Tennessee covers home care with the CHOICES in Long-term Care program, which provides benefits such as personal care and homemaker services, assistive technology, personal emergency response systems, and home modifications (23). According to a recent policy analysis, 13% of Medicare Advantage plans have been reported to offer family caregiver supports such as respite care, counseling, and skills training (24).

More than 80% of food-insecure caregivers in the sample were younger than 65 years. Approximately 64% were either unemployed or not able to work, 28% had an annual household income of $15,000 or less, and approximately 25% of informal caregivers were uninsured. Health insurance also plays an important role in food insecurity, as demonstrated by a recent study that showed a positive association between Medicaid expansion and improvement in food insecurity as a spillover effect of reducing poverty (25). Aforementioned sociodemographic characteristics of food-insecure caregivers underscore the importance of Medicaid coverage in this sample. Although the insurance programs discussed provide waivers to support caregivers, lack of health insurance or being ineligible for Medicaid in states that have not yet expanded their Medicaid programs may limit the eligibility of several caregivers to access these programs and, in turn, predispose them to food insecurity. None of the 4 states studied had expanded their Medicaid programs as of 2015. Even to date, Alabama, Mississippi, and Tennessee have not adopted Medicaid expansion. Hence, along with increasing awareness on the availability of facilities for caregivers, future health policies should focus on the development of better strategies for improving access to such services (eg, clinic-to-community models for addressing food insecurity, increased collaborations between health care systems and food assistance providers) (17). Additionally, regional variations among SNAP eligibility requirements should be streamlined to improve access to food services for caregivers who are most in need of those services (17).

Caregiving conditions often dictate the care intensity and involvement of the informal caregiver. Our study highlighted that caring for people with musculoskeletal conditions (eg, arthritis) is associated with food insecurity among caregivers. The impact of helping to manage a care recipient’s arthritis condition on a caregiver’s health-related quality of life, physical health, and mental health has been noted because of the chronic nature of the disease, which typically requires more than 20 hours of care per week (26). The demanding nature of care, along with high health care costs, could predispose caregivers to food insecurity, which underscores the need for better care coordination for patients and involvement of the caregiver in the care plan so that caregivers are aware of their financial responsibility in the situation. Further research should be conducted to understand the impact of caregiving burden on food insecurity, specifically among those caring for people with arthritis. Moreover, screening for food insecurity among caregivers of those with arthritis and other musculoskeletal conditions should be made a priority in clinical settings.

### Strengths and limitations

Our study fills a gap in the literature on food insecurity among caregivers in southern US states, where food insecurity prevalence is higher. Moreover, no previous study has examined the caregiving-related predictors of food insecurity. We used 2015 BRFSS data, and other data sets, such as the Medical Expenditure Panel Survey, have the latest food insecurity data, although their caregiver modules date back to 1998 (27). The American Community Survey also has questions on food insecurity but only asks about grandparents as caregivers and not about family caregiving, thus providing an incomplete picture of caregivers in the US (28). The National Health and Aging Trends Study (NHATS) only includes Medicare beneficiaries aged 65 years or older, and its companion, the National Study of Caregiving, is a survey of the informal caregivers of respondents in NHATS, thus limiting the generalizability of the data to the general US population (29). Noting the limitations of other data sets, we chose the BRFSS 2015 data set because of the availability of data on food insecurity and caregiving.

Our study has limitations. First, food insecurity and caregiving data were only available for the 4 southern US states of Alabama, Louisiana, Mississippi, and Tennessee, so our results may not be generalizable to the US caregiver population. Future studies should explore the caregiving-related predictors of food insecurity in a more generalizable sample. Second, we used data from 2015, and it is possible that caregiving and food insecurity prevalence have changed since then. However, food insecurity in the US has recently only minimally decreased, from 12.7% in 2015 to 10.5% in 2020, indicating that the results of this study are relevant. The COVID-19 pandemic–related unemployment (with the loss of employer-sponsored health insurance for many), income loss, and health care disruptions (increased caregiving burden if the care recipient condition worsened), coupled with the shelter-in-place policies that might have further limited access to affordable nutritious food, could have increased the risk of food insecurity among caregivers. Third, our study used self-reported questions to ascertain respondents’ food insecurity status and may be subject to social desirability and recall bias. However, this item has been validated by the US Department of Agriculture as part of their 10-item food security scale and reported to have a reliability of 0.71 (*P* < .001), helping to mitigate the risk of such biases (30). Lastly, because of the cross-sectional nature of the study, we were unable to control for temporality or to make any causal inferences. Future studies should further examine the temporal relationship between food insecurity and caregiving status.

### Conclusion

We found that food insecurity was more prevalent among caregivers compared with noncaregivers in the southern US states of Alabama, Louisiana, Mississippi, and Tennessee. These states should be considered a priority group for future food insecurity–related interventions. Our study provides insights for planning future policies focused on alleviating food insecurity among caregivers. Key strategies included timely screening in health care settings using validated food insecurity questionnaires, involvement of caregivers in care planning, helping caregivers access support services, and local food-related resources. Appropriate training, education, and support for caregivers could be incorporated into routine care settings such as physicians’ offices, hospitals, and pharmacies. Results from this study can help public health practitioners develop effective policies and direct public funds to alleviate food insecurity among caregivers.
